# Learning from ancestors

**DOI:** 10.7554/eLife.49976

**Published:** 2019-08-13

**Authors:** Suk ho Hong, Neel H Shah

**Affiliations:** Department of ChemistryColumbia UniversityNew YorkUnited States

**Keywords:** protein kinase, ancestral reconstruction, evolution, erk, extracellular regulated kinase, Human

## Abstract

Predicting ancestral sequences of protein kinases reveals the molecular details that underlie different modes of activation.

**Related research article** Sang D, Pinglay S, Wiewiora RP, Selvan ME, Lou HJ, Chodera JD, Turk BE, Gümüş ZH, Holt LJ. 2019. Ancestral reconstruction reveals mechanisms of ERK regulatory evolution. *eLife* **8**:e38805. doi: 10.7554/eLife.38805

A major goal of biological research is to understand the evolutionary histories of organisms and genes. One way to do this is to study biological entities that have become extinct, as this can provide insights into the form and function of their present-day descendants. This is perhaps best exemplified by paleogenetics and paleoproteomics research, where DNA and protein molecules from ancient biological samples are extracted and sequenced to help us better understand the evolutionary relationships between different species. Indeed, the analysis of biomolecules from our hominid ancestors has revealed significant new insights into the origins and diversity of our species (Warren, 2019). Unfortunately, DNA and proteins are degraded over time, which means that this approach can only be applied to evolutionary events from the past one million years.

Many large gene and protein families have evolved through rounds of gene duplication and functional specialization over hundreds of millions of years, which puts them beyond the reach of paleogenetics and paleoproteomics. How, then, might we use evolution to dissect the specialized properties of individual proteins in a family? One approach, called ancestral sequence reconstruction, involves using a statistical model to analyze the sequences of closely-related proteins from different organisms and generate plausible sequences for their ancestors (Hochberg and Thornton, 2017). Actual protein samples based on these sequences can then be made in the laboratory and compared to naturally occurring proteins.

Ancestral sequence reconstruction has been applied to a variety of protein families to understand, at the molecular level, how closely-related proteins have evolved distinct biochemical properties. For example, this method was previously used to examine how individual kinases – enzymes that modify other proteins through a process called phosphorylation – select different target molecules (Howard et al., 2014). Now, in eLife, the same group, led by Liam Holt at New York University (NYU) – including Dajun Sang as first author – reports on the application of ancestral sequence reconstruction to study the evolution of a subfamily of kinases called the MAP kinases (Sang et al., 2019).

Most eukaryotic organisms have hundreds of different protein kinases – humans have over 500 (Manning et al., 2002) – and many kinases need to be phosphorylated themselves in order to become active (Nolen et al., 2004). Some kinases can phosphorylate and activate themselves, through a process called autophosphorylation, while others are dependent on another kinase to be phosphorylated. Many members of the MAP kinase family autophosphorylate, but ERK1 and ERK2 (referred to as ERK1/2) cannot autophosphorylate efficiently. The molecular characteristics that prevent them from doing so were previously unknown.

Sang et al. compiled MAP kinase sequences from a variety of organisms and used ancestral reconstruction to predict the sequences of their common ancestors (Sang et al., 2019). They used standard biochemical techniques to produce proteins with the predicted sequences, and showed that the predicted common ancestor of ERK1/2 could not autophosphorylate efficiently, whereas other ancestral MAP kinases could (Figure 1). Two mutations were found when the sequence of the ERK1/2 common ancestor was compared to the sequences of the other ancestral MAP kinases. One was an amino acid substitution near the spine connecting different regions of the protein; the other was an amino acid deletion that shortened a flexible loop near the catalytic cleft in the kinases. Together, these two mutations suppress the ability of ERK1/2 and their common ancestor to autophosphorylate (Figure 1). Notably, Sang et al. – who are based at Memorial Sloan Kettering, the Icahn School of Medicine and Yale – also showed that reinserting the deleted amino acid in the flexible loop in human ERK1 relieved its dependence on other kinases for its activation in cells.

**Figure 1.**
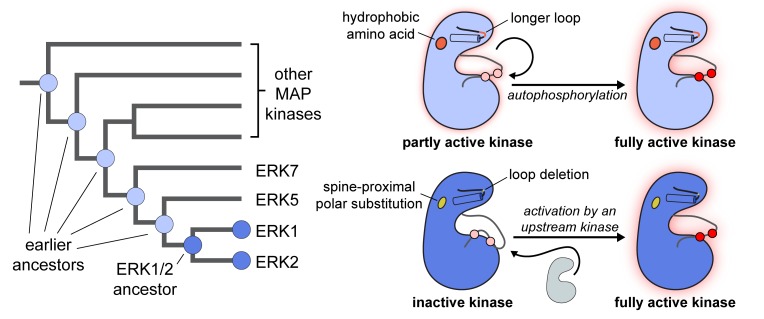
The evolution of different regulatory properties in MAP kinases. A mock phylogenetic tree (left) shows the evolution of ERK1/2 and other MAP kinases. ERK1/2, and their common ancestor (dark blue) cannot efficiently activate themselves through autophosphorylation. More ancient ancestors in the MAP kinase family (light blue) are capable of efficient autophosphorylation. A cartoon diagram (right) highlights the structural properties that differentiate MAP kinases that are capable of autophosphorylation (light blue) from those that cannot autophosphorylate themselves efficiently (dark blue). All protein kinases have a two-lobe structure with a catalytic cleft in the middle. Different parts of the kinase are connected by a spine. The loop in front of the catalytic cleft has to shift position for the enzyme to become active. This is driven by phosphorylation of that loop, either by another kinase or through autophosphorylation (shown as pink residues in the inactive form of the enzyme becoming red residues in the active form, with a concomitant change in the shape of the loop). Sang et al. have identified two mutations that could explain why ERK1/2 and their common ancestor (bottom right, dark blue) are different from other MAP kinases (top right, light blue): i) they have a polar amino acid (yellow, bottom) rather than a hydrophobic amino acid (orange, top) at a site near the spine of the kinase; ii) a loop above the catalytic cleft is one amino acid shorter than in other MAP kinases. It is thought that these two mutations disrupt the geometry and flexibility of the catalytic cleft, altering the ability of the kinase to autophosphorylate.

To explain how these evolutionary sequence alterations resulted in a change in autophosphorylation ability, Sang et al. performed computer simulations of the internal motions of ERK2, with and without the ancestral insertion and substitution. These simulations revealed that the overall flexibility of ERK2 increased when it had ancestor-like sequence features. Sang et al. postulate that increased flexibility in the mutant kinase allows it to more readily adopt a shape compatible with autophosphorylation.

The two mutations reported in the latest work have intriguing implications for kinases in general. Alterations to the flexible loop have been observed in cancer-associated variants of several distantly related kinases (BRAF, HER2, and EGFR; Foster et al., 2016), and mutations at the spine-proximal position are associated with excessive activation and drug resistance in a variety of kinases (Azam et al., 2008). Although ERK1/2 are intimately embedded within oncogenic signaling pathways, mutations at these positions have not been found in those kinases in human cancers. Further analysis of kinase evolutionary history, juxtaposed with cancer genome sequencing, is likely to reveal other conserved mutational hotspots that have facilitated the evolution of divergent properties across protein kinases.
